# Acorn Barnacles Secrete Phase‐Separating Fluid to Clear Surfaces Ahead of Cement Deposition

**DOI:** 10.1002/advs.201700762

**Published:** 2018-03-14

**Authors:** Kenan P. Fears, Beatriz Orihuela, Daniel Rittschof, Kathryn J. Wahl

**Affiliations:** ^1^ Chemistry Division Naval Research Laboratory 4555 Overlook Ave. SW Washington DC 20375 USA; ^2^ Duke University Marine Laboratory 135 Duke Marine Lab Rd Beaufort NC 28516 USA

**Keywords:** biofouling, in situ Raman microscopy, in vivo confocal microscopy, nanofibrils, protein self‐assembly

## Abstract

Marine macrofoulers (e.g., barnacles, tubeworms, mussels) create underwater adhesives capable of attaching themselves to almost any material. The difficulty in removing these organisms frustrates maritime and oceanographic communities, and fascinates biomedical and industrial communities seeking synthetic adhesives that cure and hold steadfast in aqueous environments. Protein analysis can reveal the chemical composition of natural adhesives; however, developing synthetic analogs that mimic their performance remains a challenge due to an incomplete understanding of adhesion processes. Here, it is shown that acorn barnacles (*Amphibalanus* (=*Balanus*) *amphitrite*) secrete a phase‐separating fluid ahead of growth and cement deposition. This mixture consists of a phenolic laden gelatinous phase that presents a phase rich in lipids and reactive oxygen species at the seawater interface. Nearby biofilms rapidly oxidize and lift off the surface as the secretion advances. While phenolic chemistries are ubiquitous to arthropod adhesives and cuticles, the findings demonstrate that *A. amphitrite* uses these chemistries in a complex surface‐cleaning fluid, at a substantially higher relative abundance than in its adhesive. The discovery of this critical step in underwater adhesion represents a missing link between natural and synthetic adhesives, and provides new directions for the development of environmentally friendly biofouling solutions.

Researchers have long sought to determine how marine organisms adhere to surfaces for multiple reasons. From an economic perspective, the estimated annual global cost of ship hull fouling is $180 to $260 billion USD.^1^ There are also the significant ecological impacts of increased fuel consumption, transference of non‐native species, and bioaccumulation of toxins used in antifouling coatings.^2^ From a technological perspective, fast‐curing synthetic analogs of natural marine adhesives that can be dispensed in biocompatible solvents would be extremely useful for biomedical applications (e.g. wound healing). A common approach to developing synthetic underwater adhesives is the inclusion of catechol groups,^3^, ^4^, ^5^, ^6^ such as 3,4‐dihydroxyphenyl‐l‐alanine (DOPA), due to their prevalence in natural marine adhesives and ability to displace surface water in their reduced state.^7^, ^8^, ^9^ Interestingly, barnacles secrete an adhesive that does not contain DOPA,^10^, ^11^, ^12^ yet is stronger than DOPA‐containing mussel and limpet adhesives.^13^ This disparity suggests barnacles have evolved a divergent strategy for surface adhesion that warrants further examination.

Motile barnacle larvae (cyprids) settle, metamorphose, and grow into mature barnacles on surfaces where microfouler (e.g., algae) biofilms are likely to already exist. Since barnacles are sessile, the permanence of the adhesive secreted during metamorphosis and subsequent growth cycles is paramount to their survival. Gohad et al. noted when cyprids attach there often is a void between biofilms and the adhesive plaque.^14^ This phenomenon suggests cyprids may form a barrier that protects their proteinaceous adhesive from microbial attack, possibly through the use of lipids and reactive oxygen species.^14^, ^15^ Since these chemistries are deposited ahead of new growth, they could also play a vital role in manipulating the chemical environment at the interface. Therefore, we hypothesize mature barnacles have evolved a similar process for cleaning and conditioning the interface to promote cement adhesion.

To delineate preparatory processes that aid cement adhesion, we must first clarify how and when cement is delivered to the interface. Darwin gave the earliest description of the network of ducts connected to secretory glands in barnacles (**Figure** **1**), which he termed the cementing apparatus.^16^ Walker reported cyprid cement glands dedifferentiate and redifferentiate to form the secretory glands in the cementing apparatus.^17^ Secretions in these glands positively stain for proteins, phenolic amino acids, and phenoloxidase, leading to the belief that cement is a quinone‐tanned (sclerotized) product of these secretions.^18^, ^19^ However, we note that the new secondary and circumferential ducts that form each molting cycle also undergo sclerotization; therefore, the exact role of the secretory gland proteins remains unclear. Furthermore, So et al. demonstrated that different phenolic‐modifying enzymes are present at the adhesive interface then in the region of the secretory glands.^20^ Burden et al. proposed an alternative stepwise process wherein a nonfibrillar cement secretion (BCS1) is continually released at the periphery and a fibrillar cement secretion (BCS2) is periodically released by the circumferential ducts that enhances barnacle adhesion.^21^ However, Burden et al. only tracked autofluorescence during their in vivo microscopy experiments,^21^, ^22^ which provides limited information about interfacial processes and chemistries.

**Figure 1:**
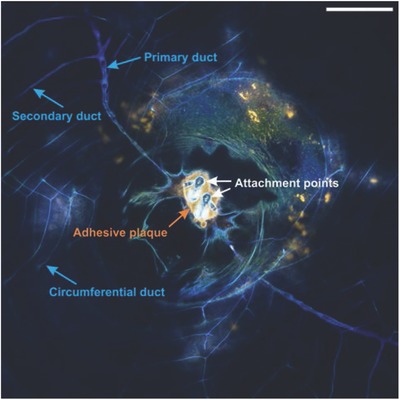
Confocal microscopy image of a barnacle after exposure to artificial seawater (ASW) containing a neutral lipid probe (Bodipy 493/503, green) and a primary amide probe (Alexa Fluor 647 NHS, orange). The image shows the cyprid attachment site (white arrows) and adhesive plaque (orange arrow), as well as part of the cementing apparatus that consists of primary ducts, secondary ducts, and circumferential ducts around the barnacle base. The cuticle and ducts exhibit strong autofluorescence on the blue channel (ex 405 nm). Scale bar represents 100 µm.

To spatially and temporally track various chemistries, in this study, we establish confocal laser scanning microscopy (CLSM) protocols for imaging live barnacles labeled with multiple fluorescent probes. This allows us to examine the basal growth zone throughout the molting cycle, which typically lasts 1 to 3 d in laboratory grown barnacles, and capture transient processes that are easily missed in ex situ imaging. In brief, cyprids were settled in glass‐bottomed Petri dishes and reared in artificial seawater (ASW). Barnacles were then transferred to ASW containing selected fluorescent probes at a minimum of two weeks after metamorphosis, and continually exposed to the probes for the duration of CLSM analysis. We note that the average growth rate of barnacles exposed to multiple fluorophores, 94 ± 23 µm per day (mean ± 95% C.I., N = 8), is comparable to healthy barnacles in nature and much faster than barnacles whose growth is stunted by environmental stresses.^23^

**Figure** **2**b–e is the representative of barnacles in the proecdysial period of their molting cycle—that is, before the body exoskeleton and basal cuticle molt. We observe epidermal cells forming a new ring of circumferential ducts (Figure 2c; Video S1, Supporting Information) that terminate at the junction between the new and the old cuticular layers (ecdysial line). The new cuticular tissue begins to sclerotize shortly before ecdysis, the point at which the body exoskeleton and basal cuticle simultaneously shed (Figure 2f,g). As the basal region expands during barnacle growth, the cuticle pulls around the leading edge and ups the side shell; cuticular folds allow the new cuticle to stretch (Figure 2), whereas the molted cuticle becomes taut and eventually rips (Figure 2m; Video S2, Supporting Information). Burden et al. concluded that a peripheral cement section (BCS1) provides some adhesion, but does not limit slip and expansion of the cuticle.^22^ Here, we can discern that protein deposits at the periphery are not secreted, per se; rather, cuticular slip results in the physical transfer of proteins and lipids to the substratum, as is shown by the streaks in Figure 2o and Video S3 (Supporting Information). We note these surface deposits often positively stain for reactive carbonyl species (i.e., aldehydes and/or ketones), meaning they are susceptible to cross‐linking to amine‐bearing materials, and phosphorylated amino acids. Furthermore, the transfer of lipids could affect the hydration state of the interface and help promote the adhesion of subsequent cement secretions.

**Figure 2:**
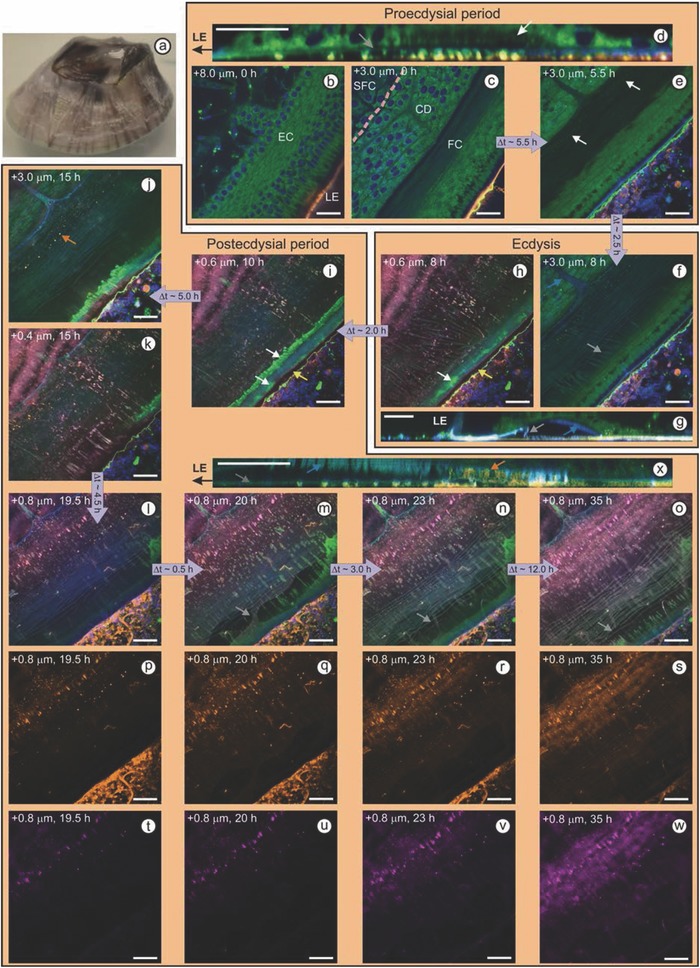
a) Photograph of an acorn barnacle. b–x) Confocal microscopy images of a barnacle in ASW containing a nucleic acid probe (DAPI, blue), a neutral lipid probe (Bodipy 493/503, green), a reactive carbonyl species probe (Alexa Fluor 555 hydrazide, magenta), and a primary amide probe (Alexa Fluor 647 NHS, orange). Distances in the top left corners correspond to height above the interface. All scale bars represent 20 µm. (b) Cuticle‐secreting epidermal cells (EC) anchored to the side shell at the leading edge (LE) of the barnacle by columnar cells. (c) Early stage of circumferential duct (CD) and folded cuticle (FC) development. Dashed line denotes the front of the base plate, which is surrounded by shell‐forming cells (SFC). (d) Orthogonal view of a 10 µm Z‐stack showing the forming FC (white arrow) above the old cuticle (gray arrow). (e) Lipid granules (white arrows) coalesce along the FC. (f) Older cuticle degrades and sheds during ecdysis (gray arrow), but remains intact and attached to the newly sclerotized cuticle (blue arrow). (g) Orthogonal view of a 20 µm Z‐stack showing the new cuticle (blue arrow) and molted cuticle (gray arrow). (h,i) Lipid secretions (white arrows) accumulate at the leading edge and are pulled outside as the barnacle expands. A nonlabeled zone is evident between the biofilm and the leading edge (yellow arrows). j,k) Change in fluorescence from green to orange indicates protein is accumulating within lipid granules; granules smear as barnacle grows. l–o) Molted cuticle tears (gray arrows) as the barnacle expands; fibrillar proteinaceous cement accumulates between cuticular layers. Secreted cement fibrils (p–s) gradually oxidize, presenting more reactive carbonyl groups over time (t–w). (x) Orthogonal view of an 8 µm Z‐stack shows epidermal cells secreting cement fibrils through unfolded sections of the new cuticle.

During the postecdysial period, we first detect possible cement secretions when protein accumulates within lipid granules (Figure 2j) that initially form in the proecdysial period (Figure 2e; Videos S1 and S2, Supporting Information). Later, protein fibrils, which make up the bulk of barnacle cement,^24^ rapidly assemble to form a band along the ecdysial line (Figure 2l and **Figure** **3**a,b) that increases in width as the cuticle expands (Figure 2m–o; Videos S2 and S4, Supporting Information). We note the tortuous morphology of the circumferential duct interior (Figure 3c) precludes the transport of protein fibrils or granules from the ducts to the basal growth zone. Moreover, there is no obvious link between the two secretions since granules are present in regions absent of fibrils. By using hexafluoroisopropanol to breakdown barnacle cement, we recently discovered cement fibrils mainly consist of proteins with conserved low complexity domains that are homologous to spider silks.^20^ The low complexity domains of intrinsically disordered proteins have been shown to drive self‐assembly into amyloid‐like fibrils,^25^, ^26^ similar to native barnacle cement.^24^ Accordingly, the accumulation of cement proteins within lipid granules could accelerate the formation of oligomers, thereby affecting the kinetics of fibril formation. However, epidermal cells ultimately mediate the formation of cement fibrils in juvenile and adult barnacles, as fibrils are only seen forming in regions were the newer cuticle is outstretched (Figure 2x; Videos S2, S4, and S5, Supporting Information).

**Figure 3:**
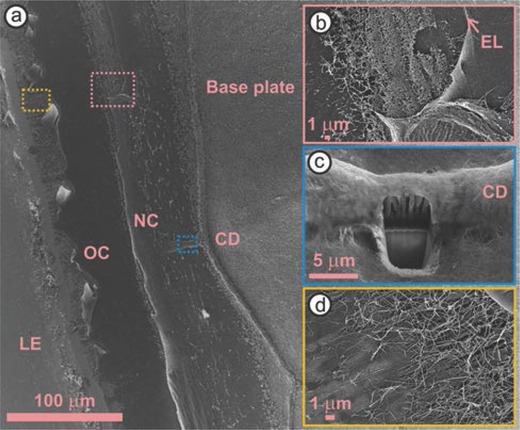
a) Scanning electron microscopy image of a barnacle attached to a substrate, after its side shell was removed. b) A band of cement nanofibrils are present along the ecdysial line (EL), between the new (NC) and old cuticle (OC) layers. c) Focused ion beam milling exposes the interior of a circumferential duct (CD). d) Cement fibrils intermix with other barnacle deposits at the leading edge (LE) under the OC.

Hydroxylation (e.g., tyrosine to DOPA) and phosphorylation (e.g., serine to phosphoserine) are post‐translational modifications commonly used by marine organisms to modify the adhesive properties of their cement proteins.^7^, ^8^, ^27^, ^28^, ^29^ As previously mentioned, barnacle cement does not contain DOPA;^10^, ^11^, ^12^ however, Gohad et al. and Dickinson et al. demonstrated that cyprid cement and the barnacle cementing apparatus are phosphorylated, respectively.^14^, ^30^ Likewise, we observe that the cyprid adhesive plaque (**Figure** **4**a), cuticular tissues, and some surface deposits positively stain for phosphorylation (Figure 4b). Conversely, newly secreted cement fibrils do not stain (Figure 4b), indicating phosphorylation does not play a direct role in the stabilization or adhesion of the fibrillar cement matrix. Instead, we observe that the amount of reactive carbonyl species present in the matrix increases over time (Figure 1t–w). While we cannot be certain if cement proteins are modified by interfacial enzymes^11^ or general oxidative stress,^35^ the formation of reactive carbonyl groups allows for covalent cross‐linking between fibrils, as well as the underlying molted cuticle, surface deposits (Figure 3d), and proteins secreted during subsequent growth cycles.

**Figure 4:**
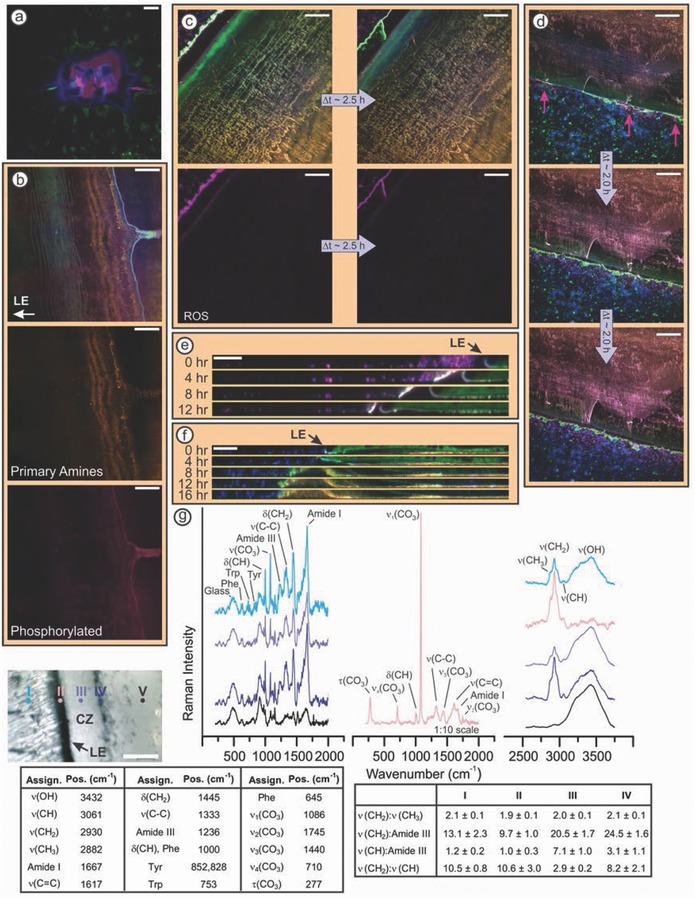
a–d) Confocal microscopy images of barnacles in ASW containing a nucleic acid probe (DAPI, blue), a neutral lipid probe (Bodipy 493/503, green), a primary amine probe (Alexa Fluor 647 NHS, orange), and either (a,b) a phosphoprotein probe (Pro Q Diamond, magenta), (c) a reactive oxygen species (ROS) probe (CellROX orange, magenta), or d,e) a reactive carbonyl species probe (Alexa Fluor 555 hydrazide, magenta). (a) Cyprid cement is heavily phosphorylated, whereas (b) barnacle cement nanofibrils are not phosphorylated. (c) Lipidaceous secretion accumulates inside the perimeter and disperses outside the perimeter. ROS are only observed after the secretion phase separates in ASW. (d) Regions of the surrounding biofilm within 10 µm of the lipidaceous secretion stain positive for reactive carbonyl species. (e) Orthogonal view of a 10 µm Z‐stack showing the removal of organic matter by the lipidaceous secretion. f) Orthogonal view of an 8 µm Z‐stack showing the clearance and subsequent protein deposition in the void under the lifted leading edge. g) Raman spectra correspond to the region marked in the optical image. The cleared zone (CZ) corresponds to the nonlabeled phase in (c) and (d). Unique bands were not observed, only varying intensities, with the exception of the calcite bands at the leading edge (region II).^31^ The position of the Amide I bands in regions I–IV (1667 cm) corresponds to β‐sheets,^32^ and agrees with previous in situ infrared spectroscopy of the adhesive interface.^33^ Tables show vibrational band assignments,^34^ and intensity ratios (*N* = 3; mean ± 95% confidence interval). All scale bars represent 20 µm.

Oxidation also plays a role in the processes *A. amphitrite* uses to mitigate fouling by microorganisms. A lipidaceous fluid accumulates at the leading edge, and infuses with the cuticle (Figure 2h,i). After the infused portion is exposed to ASW, the fluid phase separates into a nonlabeled phase, and a phase rich in lipids and reactive oxygen species (Figure 4c; Videos S1–S5, Supporting Information)—the latter of which have been shown to exhibit broad spectrum antimicrobial activity.^15^, ^36^ Organic matter within 10 µm of this secretion positively stains for reactive carbonyl species, indicating oxidation has occurred (Figure 4d). The physical properties of the secretion allow it to flow across the surface, yet it is compliant enough to displace organic matter (Figure 4e) and present a physical barrier against larger microbes (Video S6, Supporting Information). Organic matter is then carried away from the interface by the cuticular movement during basal expansion.

Dispersion of the surface‐cleaning fluid occurs even when a portion of the leading edge is not in intimate contact with the underlying substrate (Figure 4f; Video S7, Supporting Information), which would be expected to frequently occur for barnacles in their native environment. After secretion of the surface‐cleaning fluid, a protein matrix begins to fill the gelatinous phase of the fluid, sealing off the space between the barnacle and the substrate. Holm et al. demonstrated that this thick, soft adhesive plaque, known as gummy glue, has a lower tenacity than the thin, rigid plaque that normally forms.^37^ Since both plaque types consist of protein fibrils with a similar protein profile and gummy plaques are transparent when dehydrated (Figure S1, Supporting Information),^20^ differences in adhesion strength are likely due to physical properties, such as surface contact area, hydration state, and differences in the contact mechanics of the films, rather than chemical properties. Nevertheless, this observation demonstrates that the surface‐cleaning fluid has another vital function in that it facilitates the remodeling of the adhesive plaque to fill in voids beneath the barnacle and protect the adhesive interface on rough surfaces.

To obtain chemical information about the surface‐cleaning fluid, we performed in situ Raman microscopy on barnacles that were not exposed to fluorescent probes (Figure 4g). Overall, spectral features are consistent with previous reports on the chemistry of barnacle and cyprid adhesive interfaces.^14^, ^38^, ^39^ The ν(CH_2_):Amide III ratios confirms the presence of a lipid rich phase (IV) at the interface between ASW (V) and the gelatinous bulk phase of the surface‐cleaning fluid (III). Also, the slight difference (<10%) in the OH stretch intensities between ASW and the gelatinous phase indicates that this phase is well hydrated. The ν(CH):Amide III (3061:1236 cm^−1^) and ν(CH_2_):ν(CH) (2930:3061 cm^−1^) ratios reveal the gelatinous phase has a substantially higher relative abundance of phenolic chemistries than the other regions. However, the gelatinous phase lacks autofluorescence associated with quinone tanning in cuticular tissues, suggesting these chemistries may help to retain the lipidaceous phase as the secretion spreads. We note that Burden et al. detected an increase in phenolic compounds at the adhesive interface after ecdysis.^21^ While Burden et al. attributed the phenolic compounds to BCS2, the surface‐cleaning fluid is also secreted after ecdysis and could have given rise to the spectral changes they observed.

The adhesive interface of barnacles is distinctly different than the adhesive interfaces of other widely studied marine foulers (e.g., mussels and tubeworms), in particular, because they continually secrete, degrade, and recycle cuticular tissues at this interface. Since the molting process in arthropods is known to be oxidative,^40^ barnacle cement proteins must withstand oxidative stresses. More importantly, their surface adhesion must not be compromised by an oxidative environment, which explains why barnacles have evolved cement whose adhesion, unlike mussels, is not governed by the oxidation state of DOPA.^41^ The uniqueness of the surface preparation strategy used by *A. amphitrite* is currently unknown, but we know controlling the chemistry of the interface is necessary to achieve strong adhesion between any underwater adhesive, natural or synthetic, and its substratum. These findings pose the question: do other marine organisms use a similar preparatory process before cement secretion? Therefore, if we want to fully understand and mimic Nature's adhesion mechanisms, we must examine how these organisms control the adhesive interface, in addition to determining the chemical composition of their adhesives.

## Experimental Section

*Barnacle Husbandry*: Cyprids were settled on glass‐bottomed Petri dishes (Ted Pella) and reared at the Duke University Marine Laboratory for two weeks, at which point they were juvenile barnacle. Barnacles were shipped to the Naval Research Laboratory where they were placed in ASW (Instant Ocean at 32 ppt) and housed in an incubator operating at 23 °C on a 12 h day/night cycle. ASW was changed three times a week immediately before each feeding.

Barnacle used in CLSM experiments were transferred to ASW containing a nucleic acid probe (2 × 10^−6^ m DAPI), a neutral lipid probe (500 × 10^−9^ m Bodipy 493/503), a primary amide probe (200 × 10^−9^ m Alexa Fluor 647 NHS), and either an aldehyde and ketone probe (200 × 10^−9^ m Alexa Fluor 555 hydrazide), a reactive oxygen species probe (200 × 10^−9^ m CellROX orange), or a phosphoprotein probe (Pro Q Diamond). ASW solutions were exchanged daily during time lapse CLSM experiments. Stock solutions (1 × 10^−3^ m) of each probe, except Pro Q Diamond, were prepared in dimethyl sulfoxide. Since Pro Q Diamond is designed for in gel staining, the solution is rotovaped at 50 °C to remove any fixatives the stain may contain (i.e., methanol and acetic acid) and concentrate the dye, volume reduced by 75%. For staining, 4 µL of the reduced Pro Q Diamond solution was added to 10 mL of ASW. All fluorescent probes were purchased from Life Technologies (Thermo Fisher).

*Microscopy*: CLSM images were collected on a Nikon A1RSi microscope equipped with 405, 488, 561, and 640 nm lasers using the galvano scanner. Fluorescence was detected in standard mode using the A1‐DUG GaAsP multidetector unit with dichroic mirrors set at 405/488/561/640 nm. Images were collected using Plan Fluor 40 × (1.30 NA), Plan Apo λ 60 × (1.40 NA), and Apo TIRF 100 × (1.49 NA) oil immersion objectives, and laser power, gains, and thresholds were held constant for time lapse experiments. Scan parameters, including Z‐stack height and spacing, were selected to yield an acquisition time of less than 5 min per Z‐stack. Z‐stacks were collected using Nikon NIS‐Elements AR software (ver. 4.3).

Raman spectra were collected with an inVia Raman microscope (Renishaw) using a 514 nm argon ion laser. Barnacles used for Raman microscopy were not exposed to fluorescent probes. Line scans were obtained from inside the leading edge of a barnacle to the surrounding biofilm at a laser power of 15 mW over a 10 s integration period at each point.

## Conflict of Interest

The authors declare no conflict of interest.

## Supporting information

Supplementary

Supplementary

Supplementary

Supplementary

Supplementary

Supplementary

Supplementary

Supplementary
