# Supplementary material for: Acorn Barnacles Secrete Phase‐Separating Fluid to Clear Surfaces Ahead of Cement Deposition
Source: Adv Sci (Weinh). 2018 Mar 14;5(6):1700762. doi: 10.1002/advs.201700762 (PMC6010908; doi:10.1002/advs.201700762)
Supplement: Supplementary file 1 — Supplementary [file ADVS-5-1700762-s002.pdf]

Copyright WILEY-VCH Verlag GmbH & Co. KGaA, 69469 Weinheim, Germany, 2018.

ADVANCED SCIENCE
Open Access

Supporting Information

for *Adv. Sci.,* DOI: 10.1002/advs.201700762

Acorn Barnacles Secrete Phase-Separating Fluid to Clear Surfaces Ahead of Cement Deposition

Kenan P. Fears, Beatriz Orihuela, Daniel Rittschof, and Kathryn J. Wahl*

Copyright WILEY-VCH Verlag GmbH & Co. KGaA, 69469 Weinheim, Germany, 2016.

Supporting Information

Acorn barnacles secrete phase-separating surfactant mixture to clear surfaces ahead of cement deposition

Kenan P. Fears, Beatriz Orihuela, Daniel Rittschof, Kathryn J. Wahl*

Video S1. Apo TIRF 100× (1.49 NA) oil immersion objective (127.28 µm × 127.28 µm frame). Barnacle #1, 6 weeks after metamorphosis (adult), in ASW containing a nucleic acid probe (2 µM DAPI, blue, ex. 405 nm), a neutral lipid probe (500 nM Bodipy 493/503, green, ex 488 nm), a primary amide probe (200 nM Alexa Fluor 647 NHS, orange, ex 640 nm), and an aldehyde and ketone probe (200 nM Alexa Fluor 555 hydrazide, magenta, ex 561 nm). Frames collected at 15 minute intervals over a 16 hour time period.

Video S2. Apo TIRF 100× (1.49 NA) oil immersion objective (127.28 µm × 127.28 µm frame). Barnacle #2, 6 weeks after metamorphosis (adult), in ASW containing a nucleic acid probe (2 µM DAPI, blue, ex. 405 nm), a neutral lipid probe (500 nM Bodipy 493/503, green, ex 488 nm), a primary amide probe (200 nM Alexa Fluor 647 NHS, orange, ex 640 nm), and an aldehyde and ketone probe (200 nM Alexa Fluor 555 hydrazide, magenta, ex 561 nm). Frames collected at 15 minute intervals over a 30 hour time period.

Video S3. Apo TIRF 100× (1.49 NA) oil immersion objective (127.28 µm × 127.28 µm frame). Barnacle #1, 6 weeks after metamorphosis (adult), in ASW containing a nucleic acid probe (2 µM DAPI, blue, ex. 405 nm), a neutral lipid probe (500 nM Bodipy 493/503, green, ex 488 nm), a primary amide probe (200 nM Alexa Fluor 647 NHS, orange, ex 640 nm), and an aldehyde and ketone probe (200 nM Alexa Fluor 555 hydrazide, magenta, ex 561 nm). Frames collected at 15 minute intervals over a 16 hour time period.

Video S4. Plan Apo λ 60× (1.40 NA) oil immersion objective (210.35 µm × 210.35 µm frame). Barnacle #3, 2 weeks after metamorphosis (juvenile), in ASW containing a nucleic acid probe (2 µM DAPI, blue, ex. 405 nm), a neutral lipid probe (500 nM Bodipy 493/503, green, ex 488 nm), a primary amide probe (200 nM Alexa Fluor 647 NHS, orange, ex 640 nm), and a reactive oxygen species probee (200 nM CellROX orange, magenta, ex 561 nm). Frames collected at 15 minute intervals over a 24 hour time period.

Video S5. Apo TIRF 100× (1.49 NA) oil immersion objective (127.28 µm × 127.28 µm frame). Barnacle #3, 2 weeks after metamorphosis (juvenile), in ASW containing a nucleic acid probe (2 µM DAPI, blue, ex. 405 nm), a neutral lipid probe (500 nM Bodipy 493/503, green, ex 488 nm), a primary amide probe (200 nM Alexa Fluor 647 NHS, orange, ex 640 nm), and a reactive oxygen species probee (200 nM CellROX orange, magenta, ex 561 nm). Frames collected at 15 minute intervals over a 14 hour time period.

Video S6. Plan Fluor 40× (1.30 NA) oil immersion objective (318.20 µm × 318.20 µm frame). Barnacle #4, 8 weeks after metamorphosis (adult), in ASW containing a nucleic acid probe (2 µM DAPI, blue, ex. 405 nm), a neutral lipid probe (500 nM Bodipy 493/503, green, ex 488

nm), and a primary amide probe (200 nM Alexa Fluor 555 NHS, orange, ex 561 nm). Frames collected at 8 second intervals over a 3 minute time period.

Video S7. Apo TIRF 100× (1.49 NA) oil immersion objective. Four stitched frames in 2×2 pattern (248.34 µm × 248.34 µm total frame). Barnacle #5, 2 weeks after metamorphosis (juvenile), in ASW containing a nucleic acid probe (2 µM DAPI, blue, ex. 405 nm), a neutral lipid probe (500 nM Bodipy 493/503, green, ex 488 nm), and a primary amide probe (200 nM Alexa Fluor 555 NHS, red, ex 561 nm). Frames collected at 15 minute intervals over a 18 hour time period.

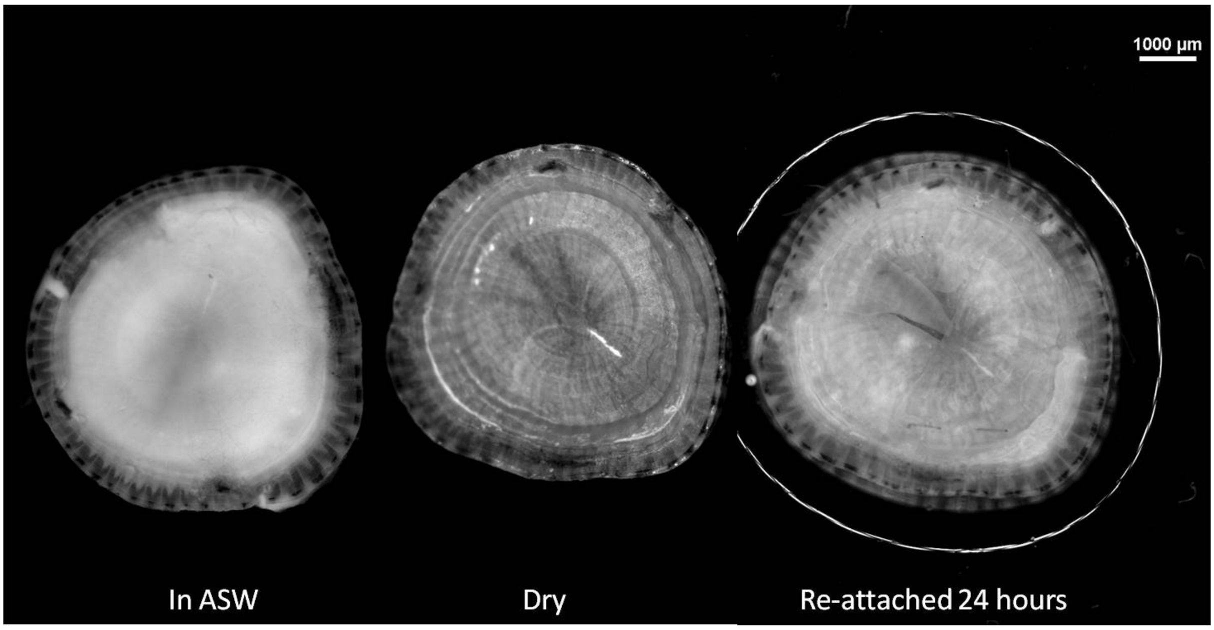

Figure S1. (Left) Barnacle with opague "gummy" glue covering most of its underside was removed from silicone panels and imaged in ASW. (Center) Image collected after the barnacle was allowed to dry in open air for 8 hours. (Right) Image collected after the dried barnacle was re-attached to a glass microscope slide and immersed in ASW for 24 hours.
